# Effect of emotional freedom technique on the fear of childbirth in Iranian primiparous women: a randomized controlled trial

**DOI:** 10.3389/fpsyg.2023.1145229

**Published:** 2024-01-08

**Authors:** Seyedeh Fatemeh Emadi, Khadijeh Hekmat, Parvin Abedi, Elham Maraghi

**Affiliations:** ^1^Midwifery Department, Reproductive Health Promotion Research Center, Ahvaz Jundishapur University of Medical Sciences, Ahvaz, Iran; ^2^Midwifery Department, Menopause Andropause Research Center, Ahvaz Jundishapur University of Medical Sciences, Ahvaz, Iran; ^3^Department of Epidemiology and Biostatistics, Ahvaz Jundishapur University of Medical Sciences, Ahvaz, Iran

**Keywords:** fear of childbirth, primiparous, emotional freedom technique (EFT), Wijma Delivery Expectancy/Experience, WDEQ-B

## Abstract

**Background:**

Fear of childbirth is one of the main causes of women’s emotional difficulty experienced in the perinatal period, especially those having their first child.

**Objective:**

The aim of this study was to investigate the effect of emotional freedom technique (EFT) on the fear of childbirth among primiparous women in Ahvaz, Iran.

**Materials and methods:**

This randomized clinical trial was conducted on 116 primiparous women. The participants were randomly divided into intervention (*n* = 58) or control (*n* = 58) groups. The intervention group received daily stimulation of certain points in their body for 12 weeks using EFT. The fear of childbirth was measured at the beginning of the study and 12 weeks after the intervention using the Wijma Delivery Expectancy/Experience Questionnaire (WDEQ-A) and at the first postpartum visit with WDEQ-B. Data were analyzed using independent *t*-test, paired *t*-test, Chi-square or Fisher’s exact test, and analysis of covariance (ANCOVA).

**Results:**

After intervention, the mean score of fear of childbirth in the intervention group decreased from 49.39 ± 8.21 to 40.42 ± 13.43 (*p* < 0.0001), while the same rate in the control group increased from 49.47 ± 9.06 to 52.09 ± 7.73 (*p* = 0.002). The mean score of fear of childbirth after delivery in the control group (45.88 ± 7.10) was higher than that in the intervention group (27.13 ± 5.08) (*p* < 0.0001).

**Conclusion:**

Based on the findings of this study, EFT can be considered as an effective method to reduce the fear of childbirth score in primiparous women.

## 1 Introduction

Although pregnancy is associated with positive feelings about the baby, it is mostly accompanied with anxiety, stress and fear of childbirth (Wijma, 2003). According to a systematic review, the prevalence of intensive fear of childbirth in women around the world is 14%, with primiparous and multiparous women accounting for 16 and 12%, respectively (O’Connell et al., 2017). In Europe, the overall prevalence of intensive fear of childbirth is 11%, which is 11.4% for primiparous women and 11% for multiparous women (Lukasse et al., 2014). In Australia, the overall prevalence of intensive fear of childbirth is 24, and 31.5% of primiparous women report severe fear (Toohill et al., 2014). Fear of childbirth has been reported differently in developing countries. For example, a study in Malawi showed that 41 and 20% of pregnant women reported moderate and high fear of childbirth, respectively (Khwepeya et al., 2018). Another study in Southern Ethiopia showed that the prevalence of fear of childbirth is 24.2%, and women with the negative experience from previous pregnancy had a higher rate of fear (Aynalem and Eneye, 2021). A study in Iran showed that 77.2% of pregnant women had mild or moderate fear of childbirth, 18.5% had clinical and 4.3% had severe fear of childbirth (Alijani et al., 2019).

Mental stress during pregnancy and childbirth can be associated with adverse outcomes and consequences such as miscarriage, nausea and vomiting, pre-eclampsia, and premature birth. Furthermore, fear of childbirth may persist in the postpartum stage and leave an unpleasant experience of childbirth (Alehagen et al., 2001). According to a previous study, preterm birth almost is twice among women who experienced stress in their pregnancy in comparison to women who did not have (Lilliecreutz et al., 2016). A longitudinal study showed that higher maternal blood leptin was significantly associated with respiratory distress, low birth weight, lower head circumference, and low APGAR score in neonates (Rabiepoor et al., 2019). Another study showed that women with higher anxiety score during pregnancy had more uterine artery resistance index that in turn can deteriorate blood flow of uterus (Fisk and Glover, 1999). In addition, the hypoxia caused by the decrease in blood flow to the pelvic muscles in response to the fear-induced increase in serum catecholamine and cortisol levels can lead to increased pain in mothers. Women who experience excessive fear during pregnancy are at risk of emotional imbalance after giving birth, which can negatively affect their relationship with their baby (Areskog et al., 1983; Lederman, 1990). Also, fear of labor pains and feeling alone during pregnancy are predictive factors of pain and suffering during childbirth and may increase the risk of emergency cesarean section (Ryding et al., 1998) and elective cesarean section (Waldenström et al., 2006). In 1985, the World Health Organization (WHO) urged that there be no justification for a cesarean rate of higher than 10–15% in any region of the world (Chalmers, 1992). The reason why the WHO recommends to avoid unnecessary cesarean section is that, like any surgery, caesarean sections are associated with short- and long-term risks which can extend many years beyond the current delivery and affect the health of the woman, her child, and future pregnancies, and that these risks are higher in women with limited access to comprehensive obstetric care” (World Health Organization, 2023).

Studies have estimated the prevalence of cesarean section to be 50–60% in Iran, which is 3–4 times higher than that recommended by the WHO (Badiee et al., 2013). Negative experiences with last delivery may persuade pregnant women to resort to elective cesarean section (Størksen et al., 2015). However, there is evidence suggesting that fear of childbirth among primiparous women is higher than in multiparous women (Shakarami et al., 2021), and that such fear is the most common reason for having a cesarean section (Negahban and Ansari, 2009) even though complications of cesarean sections are far more frequent than those of natural childbirths.

Therefore, treatment of fear of childbirth should be a priority in order to reduce the risk of having an unpleasant experience of childbirth, reduce cesarean section rate, and improve the mental health of mothers (Delavar and Alizadeh, 2014). Several methods such as hypnosis, exercise, and cognitive-behavioral interventions with or without medications have been used to reduce the fear of childbirth (O’Connell et al., 2019).

Emotional freedom technique (EFT) is one of the alternative medicine treatments which is a combination of elements of cognitive-behavioral therapy, exposure therapy, and somatic stimulation of acupuncture points (Church, 2014; Stapleton et al., 2017). Physiological examination of the clinical effects of EFT has shown that this technique is associated with the regulation of cortisol, beneficial changes in gene expression, and regulation of the autonomic nervous system activity (Church et al., 2012, 2020).

The effect of EFT has been investigated in studies in different areas. For example, Church et al. found that EFT clearly reduced the level of cortisol, which in turns improved depression, anxiety and other psychological disorders (Church et al., 2018). According to Lane, the physiological mechanisms of the response to reduced stress and relaxation include a decrease in the secretion of stress hormones such as cortisol, an increase in the secretion of endogenous opioids, and as a result the decrease of fear in the amygdala (Lane, 2009). EFT has been reported to be effective in many other areas such as the treatment of disorders such as generalized or specific anxiety, morbid fears, depression, post-traumatic stress disorder, chronic pain, and addiction (Church, 2013). However, no research has so far been conducted on the effect of EFT on the fear of childbirth in Iran. Therefore, this research was conducted with the aim of investigating the effective of EFT on the fear of childbirth among primiparous women in Ahvaz, Iran. We hypothesized that EFT could reduce the fear of childbirth among primiparous women.

## 2 Methods

This study is a parallel randomized controlled trial on 116 primiparous pregnant women with fear of childbirth who were randomly divided into EFT and control (Sham therapy) groups. The study was approved by the Ethics Committee of Ahvaz Jundishapur University of Medical Sciences (Ref. ID: IR.AJUMS.REC.1400.192). Also, the protocol of this study was registered in the Iranian Registry of Clinical Trials (Ref. ID: IRCT20210622051671N1). All participants provided written informed consent before data collection. The women were recruited in this study based on the following criteria: minimum elementary education, age 18 to 40 years, gestational age 28 to 30 weeks, primiparity, and obtaining a score greater than 37 from the Wijma Delivery Expectancy Questionnaire (WDEQ-A), which indicates moderate fear of childbirth. Women with the following criteria were excluded from the study: suffering from chronic diseases such as heart disease, high blood pressure, and diabetes, history of abortion, placenta previa, suffering from mental illnesses during the past year, taking psychoactive drugs, drug addiction, smoking, and pregnancy complications such as pre-eclampsia, hemorrhage, intrauterine death, and premature birth.

Data collection was started in November 2021 and completed in March 2022.

### 2.1 Sample size

According to the objectives of the research and assuming a power of 80%, *α* = 0.05, s1 = s2 = 17.43 and *d* = 10, the sample size was determined using the following formula:


$$n=\frac{{\left(Z_{1-\frac{\alpha }{2}}+Z_{1-\beta }\right)}^{2}\left(s_{1}^{2}+s_{2}^{2}\right)}{{\left(d\right)}^{2}}$$

Assuming a 20% attrition rate, the number of participants in each group of intervention and Sham therapy was 58.

### 2.2 Randomization

Participants who scored above 37 in the WDEQ-A were included in the study. The method of assigning women to the intervention or control groups was based on block randomization with a block size of 6 and an allocation ratio of 1:1. The randomization list was prepared by a statistician (EM). The codes assigned to the eligible women were placed in sealed envelopes by someone who was not involved in the study and was not aware of the research objectives. The envelops were then given to the clerk in health centers. In this way, neither the researcher nor the participants were aware of group allocation until the commencement of the study. This study was single blind, meaning that the researcher was aware of which group received the intervention, but participants were not aware of grouping.

### 2.3 Setting

The lead researcher visited two public health centers in Ahvaz city and screened pregnant women based on the inclusion/exclusion criteria. A total of 200 women were examined, of whom 116 who met the inclusion criteria were randomly assigned into EFT and control groups. Primiparous women who attended to Public Health Centers No. 2 and 3 in the west of Ahvaz were screened based on the inclusion criteria. Women who met inclusion criteria and received a score more than 37 from Wijma-A questionnaire were requested to fill-out the demographic questionnaire.

### 2.4 Instruments

In this study, a demographic questionnaire and the Wijma Delivery Expectancy/Experience Questionnaire (W-DEQ) versions A and B were used to collect the data. The demographic questionnaire contained questions about age, gestational age, the woman’s and her husband’s educational attainment and occupation, and economic status.

W-DEQ-A is a questionnaire used to assess women’s fear of the childbirth. This questionnaire has 33 questions in six dimensions of “lack of positive anticipation (questions number 2, 19, 25 and 27),” “fear (questions number 3, 6, 7, 8, 11, 12 and 15),” “lack of self-efficacy (questions number 4, 5, 10, 13, 14, 16, 17, 20, 22, 23),” “loneliness (questions number 1, 9 and 29),” “concerns for the child (questions number 30, 32, 33),” and “negative appraisal (questions number 18, 21, 26, 28).” Questions 24 and 31 are not classified in any category, but they are taken into account when calculating the total score. The questions are scored based a six-point Likert scale (from 0 to 5). Some of the questions are affirmatively worded and some are negatively worded. The minimum total score obtained is 0 and the maximum score is 165. A score less than or equal to 37 means a mild fear level, a score of 38–65 suggest a moderate fear, and a score of 66–84 represents severe fear. The cut-off score is 85, which means that scores 85 and above prove clinical fear (Wjma et al., 1998).

The construct validity of this questionnaire and its reliability were confirmed in Abedi et al.’s study (Abedi et al., 2016). WDEQ-B has 33 questions in six sub-scales, and each question is scored from 0 to 5. It is similar to the A version in terms of scoring and cut-off point. They are different only in terms of the time point of assessment (the A version is used before delivery while the B version is used after delivery).

### 2.5 Intervention

The EFT included the following steps:

1. Identifying the problem that caused the person to be upset (e.g., fear of childbirth) and scoring or measuring the severity of that problem.
2. Expressing an emphatic sentence (in relation to the person’s problem and self-acceptance) along with tapping the parts of the face and body with the tips of the fingers, which is known as correction (i.e., arrangement of the energy system).

**Figure 1:**
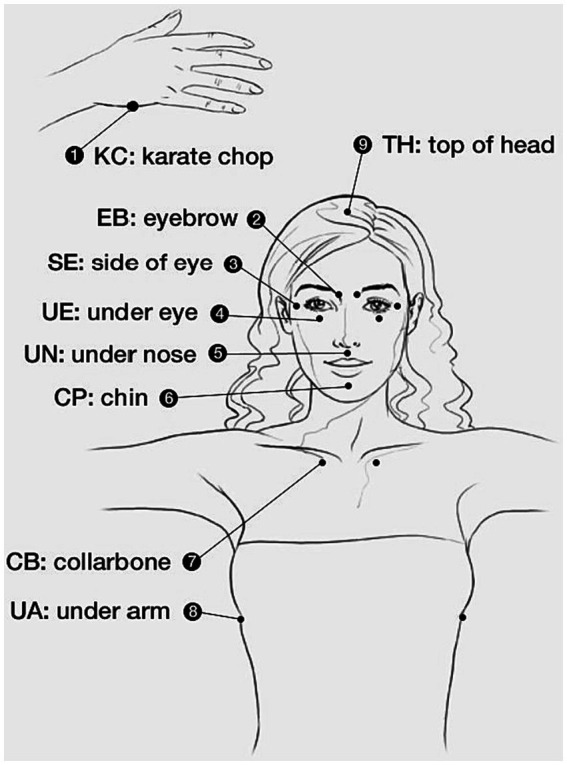
EFT points.

These points include: the edge of the hand (the fleshy part of the outer edge of the hand that is used in karate to break objects), the beginning of the eyebrows (above the side of the nose), the side of the eyes (on the bone of the outer side of the eye), under the eyes (on the cheek and 2–3 cm below the eyeball), under the nose (in the short distance between the nose and the upper lip), chin (the distance between the depression of the chin and the lower lip), the beginning of the clavicle (at the intersection of the sternum, the clavicle and the first rib, 2.5 cm towards the navel from the U-shaped slit) and under the arm (10 cm from the armpit to the bottom), above the head (if an imaginary line is drawn from one ear to the other ear and another from the nose to the back of the neck, this point will be right at the intersection of these two lines; Figure 1).

In order to learn and master the principles of EFT, the lead researcher participated in a workshop held by an expert in EFT.

In groups of maximum 10 women, the participants in the intervention group received EFT training on how to manipulate the correct points in one session. In this training session, this technique was illustrated using a short training video, and the researcher ensured the correctness of manipulating the points (this training video was prepared by the training institute).^1^ Also, an approved educational brochure (to help the participants in the correctly use of this method) which was based on the standard instructions and EFT website was provided to the participants to use at home. Based on the initial research design, two face-to-face training sessions were supposed to be held, but due to the start of the COVID-19 pandemic and the subsequent home quarantine, only one face-to-face session was held for the participants.

The participants were recommended to do this technique every morning in order to improve their mood throughout the day. In addition, they were advised to use this technique during the day whenever they felt stressed out and afraid. While tapping, the women were asked to use positive statements and to accept themselves despite all the problems they had. In the next 10 weeks, the participants were followed up (on a weekly basis) by phone calls in order to check the regular implementation of this method (i.e., sending audio files on tapping techniques three times a week and answering the mothers’ questions) in accordance with the principles taught in the face-to-face training session. The participants’ questions were answered through phone calls and text messages. The women were asked to use affirmative sentences when tapping the EFT points.

Likewise, a face-to-face training session was held for the control group, but it included false points (i.e., elbow bumps, chin bumps, eye bumps, nose bumps, foreheads, and arms). All participants were asked to follow these instructions until the end of pregnancy. In order to abide by ethical consideration, women in the control group were given a pamphlet about right points at the end of the study.

### 2.6 Follow up

We recruited women at 28–30 gestational age and followed them up for 10 weeks for measuring fear of childbirth before delivery. Women also followed up to 2 weeks after delivery for measuring fear of childbirth after delivery. Women in the intervention group received a phone call three times a week to ensure the correct use of EFT.

In the last prenatal visit (39–40 weeks), the fear of childbirth after intervention was assessed using WDEQ-A. For measuring fear of childbirth after delivery the WDEQ-B was given to the mothers (both as Google doc file and hard copy) to be completed at home after delivery. In the first postpartum visit (first 10 days after delivery) the completed questionnaires were checked by the lead researcher and in case of any unanswered question, the mothers were requested to provide answers to those questions.

### 2.7 Outcomes

- Fear of childbirth before delivery.
- Fear of childbirth after delivery.

### 2.8 Statistical analysis

Data analysis was done using SPSS version 20. The quantitative variables were reported using mean and standard deviation, and qualitative variables, using frequency (percentage). The normality of quantitative variables was assessed by the Shapiro–Wilk test. The comparison of qualitative variables in the two groups was done by Chi-square or Fisher’s exact tests. Comparison of the quantitative variables between two groups was performed using the independent samples *t*-test. Paired samples *t*-test was used for comparing the quantitative variables’ changes in each group. Effect of intervention on posttest outcome measures were examined using analysis of covariance (ANCOVA), adjusting for pretest scores. The significance level of the tests was considered as 0.05.

## 3 Results

**Figure 2:**
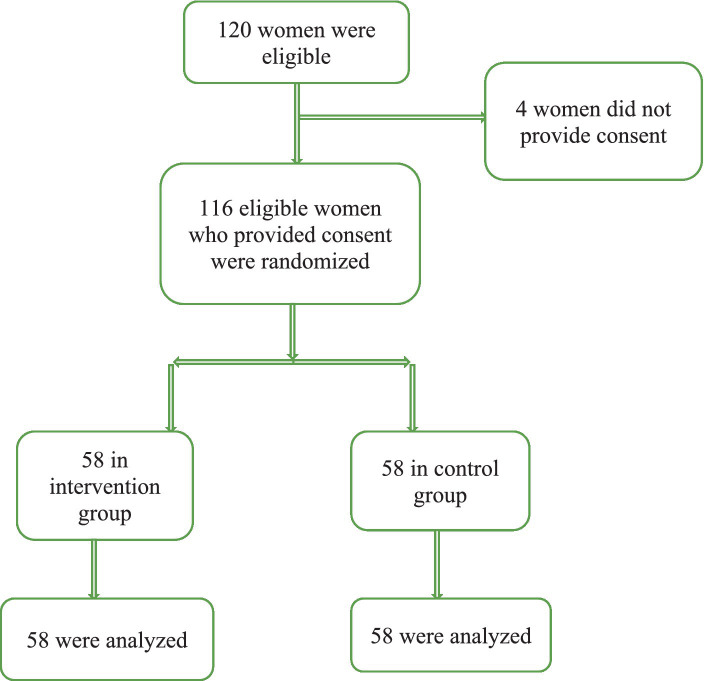
Flow-diagram of recruitment and retention of participants in the study.

In this study, 58 women were included in each group (intervention and control) and all of them completed the study (Figure 2). The demographic characteristics of the participants are listed in Table 1. The mean age of the mothers was 22.11 ± 3.73 years, and their mean gestational age was 29.15 ± 0.77 weeks. According to the results of Table 1, the two groups had no statistically significant differences in terms of demographic factors (*p* > 0.05).

**Table 1:** Demographic characteristics of the studied women in the intervention and control groups.

Variables		Mean ± SD				Value of *p*
		Intervention (*n* = 58)	Control (*n* = 58)	Statistics	*df*	
Mother’s age (year)		22.14 ± 3.70	22.06 ± 3.78	−0.12	120	0.904^*^
Gestational age (week)		29.13 ± 0.76	29.16 ± 0.77	0.23	120	0.815^*^
		*N* (%)				
Educational attainment	High school	20 (32.8)	27 (44.3)	2.68	2	0.261^**^
	High school diploma	37 (60.7)	28 (45.9)			
	University degree	4 (6.6)	6 (9.8)			
Husband’s educational attainment	High school	8 (13.1)	17 (27.9)	6.64	3	0.084^**^
	High school diploma	51 (83.6)	39 (63.9)			
	University degree	1 (1.6)	4 (6.6)			
Occupation	Housewife	57 (93.4)	57 (93.4)	0.00	1	>0.99^**^
	Employed	4 (6.6)	4 (6.6)			
Husband’s occupation	Office clerk	16 (26.2)	16 (26.2)	0.00	1	>0.99^**^
	Self-employed	45 (73.8)	45 (73.8)			
Does your income cover your expenses	Not at all	1 (1.6)	0 (0)	3.48	2	0.175^**^
	Moderately	55 (90.2)	50 (82.0)			
	Good	5 (8.2)	11 (18.0)			

^*^Independent *t*-test or Man–Whitney test. ^**^Chi-square test or Fisher exact test.

Table 2 shows the difference between the mean score of the components of WDEQ-A before and after intervention in the intervention and control groups. The mean score of lack of positive anticipation in the intervention group decreased from 7.72 ± 2.43 before the intervention to 6.86 ± 2.75 after intervention, which was not significantly different compared to the control group (*p* = 0.34). The mean score of fear in the intervention group fell from 13.60 ± 2.28 before the intervention to 10.95 ± 5.74 after intervention (*p* = 0.001), which was significantly different compared with the control group (*p* = 0.003). The mean score of lack of self-efficacy in both groups increased significantly. Results of independent *t*-test and ANCOVA did not show any significant difference between the two groups in this regard (*p* = 0.222). The mean score of loneliness in the intervention group rose from 3.08 ± 1.12 to 3.42 ± 2.51, which was not significantly different compared to the control group (*p* = 0.508). The mean score of concerns for the child in the intervention group increased from 1.36 ± 7.39 to 3.86 ± 1.46 (*p* < 0.0001), which showed a significant difference between the two groups based on independent *t*-test and ANCOVA (*p* < 0001). Finally, the mean score of negative appraisals before intervention in the control and intervention groups was 4.49 ± 3.95 and 3.31 ± 2.32, respectively (*p* = 0.001), which changed to 4.75 ± 1.61 and 3.13 ± 3.44, respectively after intervention (*p* = 0.001). Despite the lack of significant changes within the groups, the difference between the two groups was significant using ANCOVA test (*p* = 0.007). The mean overall score of fear of childbirth in the intervention group decreased from 49.39 ± 8.21 to 40.42 ± 13.43 (*p* < 0.0001), while the mean overall score of fear of childbirth in the control group increased from 49.47 ± 9.06 to 52.09 ± 7.73 (*p* = 0.002).

**Table 2:** Comparison of the mean score of the dimensions of WDEQ-A before and after intervention in the intervention and control groups.

Studied variables	Intervention (*n* = 58)	Control (*n* = 58)	value of p^*^	*t*(*df*)	*p* –value^***^	Statistic (*df*)
**Mean ± SD**						
*Lack of positive anticipation*						
Before	7.72 ± 2.43	7.21 ± 2.46	0.254	−1.14 (120)	0.060	3.54 (1)
After	6.86 ± 2.75	7.29 ± 2.11	0.340	0.95 (120)		
*p* ^**^	0.032	0.642				
*t*(*df*)	2.20 (60)	−0.46 (60)				
*Fear*						
Before	13.60 ± 2.28	13.01 ± 2.37	0.164	−1.39 (120)	0.003	8.80 (1)
After	10.95 ± 5.74	12.85 ± 2.04	0.016	2.43 (74.98)		8.80 (1)
*p* ^**^	0.001	0.448				
*t*(*df*)	3.58 (60)	0.76 (60)				
*Lack of self-efficacy*						
Before	10.52 ± 3.63	10.55 ± 3.18	0.958	0.053 (120)	0.222	1.41 (1)
After	13.47 ± 8.32	12.11 ± 2.87	0.230	−1.20 (74.12)		
*p* ^**^	0.019	<0.0001				
*t*(*df*)	−2.41 (60)	−0.76 (60)				
*Loneliness*						
Before	3.08 ± 1.12	3.22 ± 1.17	0.481	0.70 (120)	0.588	0.29 (1)
After	3.43 ± 2.51	3.65 ± 0.96	0.508	0.66 (77.20)		
*p* ^**^	0.338	<0.0001				
*t*(*df*)	−0.96 (20)	−4.79 (60)				
*Concerns for the child*						
Before	7.39 ± 1.36	7.52 ± 1.47	0.612	0.50 (120)	<0.0001	169.74 (1)
After	3.86 ± 1.46	7.78 ± 2.11	<0.0001	11.89 (106.86)		
*p* ^**^	<0.0001	0.343				
*t*(*df*)	21.62 (60)	−0.95 (60)				
*Negative appraisal*						
Before	3.31 ± 2.32	4.49 ± 3.95	0.001	0.05 (120)	0.007	39.40 (1)
After	3.13 ± 3.44	4.75 ± 1.61	0.001	5.88 (95.82)		
*p* ^**^	0.742	0.562				
*t*(*df*)	0.33 (60)	−0.58 (60)				
*The overall score*						
Before	49.39 ± 8.21	49.47 ± 9.06	0.958	0.05 (120)	<0.0001	39.40 (1)
After	40.42 ± 13.43	52.09 ± 7.73	<0.0001	5.88 (95.82)		
*p* ^**^	<0.0001	0.002				
*t*(*df*)	4.67 (60)	−3.32 (60)				

The fear of childbirth was measured before intervention (weeks 28–30) and after intervention (weeks 39–40) of pregnancy. ^*^Independent *t*-test; ^**^Paired *t*-test; ^***^ANCOVA.

Table 3 shows the difference in the mean score of the dimensions of WDEQ-B after delivery in the intervention and control groups. In all dimensions of fear of childbirth including lack of positive anticipation, fear, lack of self-efficacy, loneliness, concerns for the child, except negative appraisal, the score of the intervention group was significantly lower than that of the control group (*p* < 0.0001). The mean overall score of fear of childbirth after delivery in the control and intervention groups was 45.88 ± 7.10 and 27.13 ± 5.08, respectively, indicating a significant difference (*p* < 0.0001). No side effects were reported by participants in intervention or control groups.

**Table 3:** Comparison of the mean score of the dimensions of WDEQ-B in the intervention and control groups.

Studied variables	Intervention (*n* = 58)	Control (*n* = 58)	*t* (*df*)	Value of p^*^
**Mean ± SD**				
Lack of positive anticipation	6.96 ± 1.19	8.62 ± 1.08	8.01 (120)	<0.0001
Fear	10.18 ± 1.61	18.45 ± 3.31	17.51 (86.97)	<0.0001
Lack of self-efficacy	0.77 ± 1.10	8.31 ± 2.04	25.35 (92.09)	<0.0001
Loneliness	0.49 ± 0.84	3.29 ± 1.08	15.88 (113.42)	<0.0001
Concerns for the child	3.77 ± 1.08	2.34 ± 1.15	−7.03 (120)	<0.0001
Negative appraisal	0.42 ± 0.97	0.13 ± 0.78	−1.84 (114.80)	0.068
Overall score	27.13 ± 5.08	45.88 ± 7.10	16.77 (108.70)	<0.0001

Fear of childbirth after delivery was measured up to two weeks after delivery. ^*^Independent *t*-test.

## 4 Discussion

This study was conducted to investigate the effect of EFT on the fear of childbirth in primiparous women. The results showed that the mean score of fear, concerns for the child, negative appraisal, and the overall score of Wijma-A were significantly improved in the intervention group compared to the control group. However, the two groups were not significantly different regarding lack of positive anticipation, lack of self-efficacy, and loneliness. The scores of all dimensions of Wijma-B except for negative appraisal were improved significantly in the intervention group compared to the control group.

EFT has been successfully used in treatment of various mental and physical disorders including depression, anxiety, phobias, post-traumatic stress disorder, irritable bowel syndrome, and obesity, proving to be effective usually within 1–10 sessions (Wells et al., 2003; Baker and Siegel, 2010). We could not find any study to investigate the effect of EFT on the dimensions of W-DEQ, but there are a number of studies that evaluated the effect of EFT on the stress, anxiety and fear of expectant mothers.

In a study on 120 pregnant women, for instance, Vural et al. investigated the role of EFT and conscious breathing in reducing fear of childbirth. They used the Subjective Units of Distress Scale and Wijma-B for measuring fear of childbirth. Their results showed that both types of intervention, namely EFT and conscious breathing, reduced the level of fear of childbirth, but the former had more profound and permanent effects (Vural and Aslan, 2019). Another study by Mardjan et al. was conducted in 2016 in Indonesia on 38 teenage pregnant women suffering from anxiety. Their study was aimed at investigating the effect of EFT on anxiety and cortisol levels, and they used Taylor Manifest Anxiety Scale (TMAS) and cortisol blood test for measuring anxiety level. Their results showed that EFT significantly contributes to the reduction of anxiety level and blood cortisol level by 57 and 43%, respectively, and that it indirectly affects the mother’s readiness for childbirth (Mardjan et al., 2018). In 2015, in a semi-quasi study with a pre-test-post-test design and control group in Tehran, Ghamsari et al. investigated the effect of EFT on perceived stress and tolerance of 30 pregnant women. They found that the experimental group’s scores on perceived stress significantly decreased while there was a significant increase in their scores of tolerance (Ghamsari and Lavasani, 2015). In Yuniarti et al. (2016) investigated the effect of EFT on the level of cortisol, immunoglobulin E, and the anxiety among pregnant women. Primiparous women who used this technique were more relaxed when facing childbirth (Yuniarti et al., 2016). Our results are in line with the above studies. The exact mechanism for stress and fear reduction of EFT is not clear, but evidence showed that improvement in physical parameters such as heart rate variability, hearth coherence, blood pressure, and level of cortisol, that all of them are predisposing factors for stress and anxiety, may play a main role in stress and fear reduction (Bach et al., 2019). Also, as EFT has almost similar mechanism to mindfulness, one of the underlying factors of stress reduction of both techniques is attention to present moments and monitor and acceptance (Lindsay et al., 2018).

Studies have shown that a high level of fear of childbirth before childbirth can persist for a long time after childbirth and that such fear is associated with the development of symptoms of post-traumatic stress disorder (Wijma et al., 1997). In their study on the fear of childbirth before, during and after childbirth, Alehagen et al. found that women with fear before childbirth are more likely to have fears during the active stage of labor and after childbirth (Alehagen et al., 2006). Screening to identify a high level of fear of childbirth during pregnancy can provide appropriate treatment for women with a high level of fear, possibly by reducing the risk of negative consequences in the future, both psychologically and obstetrically (Zar et al., 2001). Evidence from our study show that using EFT in the third trimester could reduce the fear of childbirth in postpartum women.

In Iran systematic efforts are being made in health centers to minimize the anxiety and fear of primiparous mothers, including counseling and pregnancy exercise under the supervision and guidance of an authorized midwife (Baker and Siegel, 2010). Of course, this sometimes makes it difficult for the mother to arrange her time to go to health centers and receive services (Roland-Price and Chamberlain, 2002). EFT enables mothers to do this technique either under the supervision of a teacher or independently at home without time limits and with the best possible results (Baker and Siegel, 2010).

Although, in the present study, the intervention based on EFT improved fear, concerns for the child, negative appraisal, and the overall score of Wijma-A, it failed to improve scores in the scales “lack of positive anticipation,” “lack of self-efficacy,” and “loneliness”. Satisfaction with childbirth is related to a variety of psychological factors which include, but are not limited to, sense of personal control and whether health providers and family are meeting the women’s expectations (Goodman et al., 2004). Therefore, it is likely that the EFT could not improve all these dimensions. Furthermore, the participants in the present study were primiparous and may had deeper fear of childbirth compared to women who already had other pregnancies, and this might have hindered their ability to overcome to any unanticipated situation happened around the time of labour and to experience a sense of self-efficacy making the improvement of the scales ‘lack of positive anticipation’ and ‘lack of self-efficacy difficult. Finally, physical and mental changes that occur during pregnancy might alienate a person from herself and the society around her (Rokach, 2004), and, although EFT could reduce feeling of loneliness to some extent, the changes did not reach a significant level.

The hypothesis of this study that EFT could significantly reduce the fear of childbirth was confirmed by our results. The results of this study can therefore be used by policy makers and health providers to improve the mental condition of primiparous women and to improve the quality of childbirth preparation classes.

### 4.1 Strengths and limitations

This study has some strengths. First, it included a rigorous randomization of the sample in the two groups which eliminated selection bias and a design that allowed direct comparison between the two interventions. Second, a perspective design which assessed fear of childbirth before and after delivery (not only before it). Third, an adequate sample size was used in this study which was *a priori* calculated performing a power analysis. Finally, allocation to either group was not disclosed to participants, and interventions were similar (using sham therapy in the control group) enough to minimize the performance bias in women, and groups were comparable in terms of socio-demographic characteristics.

However, this study was not without limitations. First, we did not measure the cortisol level before and after the intervention. If we had, we could have come up with a better understanding of the role of EFT. Second, due to the spread of COVID-19 and the subsequent home quarantine, we were able to hold only one face-to-face training session. Third, the psychological effect of COVID-19 along with the home quarantine was not measured as a confounding factor affecting the women’s mental state and level of fear and anxiety. Fourth, recruitment was done only in two centers which might have created a selection bias in terms of participants’ education, age, and SES. Finally, the exclusion and inclusion criteria limited the study population. Therefore, future studies are recommended to replicate this study with a larger and more diverse population. Based on the strengths and limitations of the present study, further studies are advised to measure the cortisol level, rule out other causes of psychological disorders such as anxiety and depression, or be conducted in a multi-center design.

## 5 Conclusion

The results of this study showed that a 12-week EFT program can reduce the mean score of fear of childbirth in primiparous Iranian women, and this reduction is maintained even after delivery. It is recommended to use this technique in childbirth preparation classes to improve the mental health of these mothers.

## Data availability statement

The raw data supporting the conclusions of this article will be made available by the authors, without undue reservation.

## Ethics statement

The studies involving humans were approved by Ahvaz Jundishapur University of Medical Sciences. The studies were conducted in accordance with the local legislation and institutional requirements. The participants provided their written informed consent to participate in this study.

## Author contributions

SE, KH, PA, and EM were contributed to design of the study, equally contributed to data analyzing, and interpretation. SE collected the data. SE and PA prepared the first draft of the manuscript. All authors contributed to the article and approved the submitted version.

## Footnotes

^1^www.eftbylili.com, www.emofree.com
